# Initiating angiotensin II at lower vasopressor doses in vasodilatory shock: an exploratory post-hoc analysis of the ATHOS-3 clinical trial

**DOI:** 10.1186/s13054-023-04446-1

**Published:** 2023-05-05

**Authors:** Patrick M. Wieruszewski, Rinaldo Bellomo, Laurence W. Busse, Kealy R. Ham, Alexander Zarbock, Ashish K. Khanna, Adam M. Deane, Marlies Ostermann, Richard G. Wunderink, David W. Boldt, Stew Kroll, Chuck R. Greenfeld, Tony Hodges, Jonathan H. Chow, the Angiotensin II for the Treatment of High-Output Shock 3 (ATHOS-3) Investigators

**Affiliations:** 1 Departments of Pharmacy and Anesthesiology, Mayo Clinic, Rochester, MN USA; 2 Department of Critical Care, Melbourne Medical School, University of Melbourne, Parkville, Australia; 3 Australian and New Zealand Intensive Care Research Centre, School of Public Health and Preventive Medicine, Monash University, Melbourne, VIC Australia; 4 Department of Medicine, Emory University, Atlanta, GA USA; 5 Emory Critical Care Center, Emory Healthcare, Atlanta, GA USA; 6 Department of Critical Care Medicine, Mayo Clinic Arizona, Phoenix, AZ USA; 7 Department of Anesthesiology, Intensive Care and Pain Medicine, University Hospital Münster, University Münster, Munster, Germany; 8 Department of Anesthesiology, Section on Critical Care Medicine, Wake Forest School of Medicine, Wake Forest Baptist Medical Center, Winston-Salem, NC USA; 9 Perioperative Outcomes and Informatics Collaborative, Winston-Salem, NC USA; 10 Outcomes Research Consortium, Cleveland, OH USA; 11 Department of Critical Care, King’s College London, Guy’s and St Thomas’ Hospital, Westminster Bridge Road, London, SE1 7EH UK; 12 Division of Pulmonary and Critical Care Medicine, Department of Medicine, Feinberg School of Medicine, Northwestern University, Chicago, IL USA; 13 Department of Anesthesiology and Critical Care Medicine, University of California Los Angeles, Los Angeles, CA USA; 14 La Jolla Pharmaceutical Company, Waltham, MA USA; 15 Department of Anesthesiology and Critical Care Medicine, George Washington University School of Medicine and Health Sciences, 2700 M St. NW, 7Th Floor, Room 709, Washington, DC 20037 USA

**Keywords:** Angiotensin, Shock, Vasopressor, Catecholamine, Multimodal, Outcomes

## Abstract

**Background:**

High dose vasopressors portend poor outcome in vasodilatory shock. We aimed to evaluate the impact of baseline vasopressor dose on outcomes in patients treated with angiotensin II (AT II).

**Methods:**

Exploratory post-hoc analysis of the Angiotensin II for the Treatment of High-Output Shock (ATHOS-3) trial data. The ATHOS-3 trial randomized 321 patients with vasodilatory shock, who remained hypotensive (mean arterial pressure of 55–70 mmHg) despite receiving standard of care vasopressor support at a norepinephrine-equivalent dose (NED) > 0.2 µg/kg/min, to receive AT II or placebo, both in addition to standard of care vasopressors. Patients were grouped into low (≤ 0.25 µg/kg/min; n = 104) or high (> 0.25 µg/kg/min; n = 217) NED at the time of study drug initiation. The primary outcome was the difference in 28-day survival between the AT II and placebo subgroups in those with a baseline NED ≤ 0.25 µg/kg/min at the time of study drug initiation.

**Results:**

Of 321 patients, the median baseline NED in the low-NED subgroup was similar in the AT II (n = 56) and placebo (n = 48) groups (median of each arm 0.21 µg/kg/min, *p* = 0.45). In the high-NED subgroup, the median baseline NEDs were also similar (0.47 µg/kg/min AT II group, n = 107 vs. 0.45 µg/kg/min placebo group, n = 110, *p* = 0.75). After adjusting for severity of illness, those randomized to AT II in the low-NED subgroup were half as likely to die at 28-days compared to placebo (HR 0.509; 95% CI 0.274–0.945, *p* = 0.03). No differences in 28-day survival between AT II and placebo groups were found in the high-NED subgroup (HR 0.933; 95% CI 0.644–1.350, *p* = 0.71). Serious adverse events were less frequent in the low-NED AT II subgroup compared to the placebo low-NED subgroup, though differences were not statistically significant, and were comparable in the high-NED subgroups.

**Conclusions:**

This exploratory post-hoc analysis of phase 3 clinical trial data suggests a potential benefit of AT II introduction at lower doses of other vasopressor agents. These data may inform design of a prospective trial.

*Trial registration*: The ATHOS-3 trial was registered in the clinicaltrials.gov repository (no. NCT02338843). Registered 14 January 2015.

**Supplementary Information:**

The online version contains supplementary material available at 10.1186/s13054-023-04446-1.

## Introduction

Vasodilatory shock is the most common form of shock and can result in high rates of organ failure and death [1]. Even brief periods of hypotension are associated with risk of renal injury, myocardial injury, and death, an effect that is exacerbated by the duration and severity of hypotension [2, 3]. Patients with persistent hypotension despite fluid resuscitation are treated with catecholamines and vasopressin with the goal of achieving a mean arterial pressure (MAP) ≥ 65 mmHg [4]. However, these agents alone may be inadequate to attain hemodynamic goals [5], suggesting the need for additional agents with alternative mechanisms of action.

Angiotensin II (AT II) is an endogenous peptide hormone and a component of the renin–angiotensin–aldosterone system (RAAS). This compound has a unique mechanism of action, distinct from those of catecholamines and vasopressin [6]. The Angiotensin II for the Treatment of High Output Shock (ATHOS-3) trial randomized patients requiring > 0.2 µg/kg/min of norepinephrine-equivalent dose (NED) to either synthetic AT II or placebo and demonstrated a difference in the primary outcome of blood pressure response with a significantly higher proportion of patients meeting their hemodynamic goals after receiving AT II compared to placebo [7]. Despite not being designed to detect a difference in mortality, several pre-specified and post-hoc analyses of the ATHOS-3 trial have reported improvements in survival in relevant subsets of patients [8–11].

Despite these observations, in the real-world setting, AT II has been initiated at much higher baseline vasopressor doses (i.e., > 0.5 µg/kg/min) than doses used in the ATHOS-3 trial (> 0.2 µg/kg/min), often as ‘salvage therapy’ leading to suboptimal outcomes [12–15]. Because of the poor prognosis associated with high-dose vasopressor support, along with the potential benefits of early multimodal use of combined vasopressors, we performed an exploratory post-hoc analysis of the ATHOS-3 trial data to investigate the impact of baseline vasopressor dose at the time of AT II initiation on patient outcomes. We hypothesized that initiation of AT II administration at lower vasopressor doses would be associated with improved hemodynamic response and 28-day survival compared with placebo.

## Methods

### Study design

The design of ATHOS-3 has been previously reported [7, 16]. Briefly, this phase 3 trial was an international, double-blind, randomized controlled trial of 321 patients with vasodilatory shock (defined as hypotension with a cardiac index > 2.3 L/min/m^2^ or with central venous oxygen saturation > 70%, and central venous pressure > 8 mmHg) receiving vasopressor support. Patients were randomized to either treatment with AT II plus standard of care or placebo plus standard of care. The primary endpoint was ability to achieve a target MAP response, defined as MAP of ≥ 75 mmHg or an increase of at least 10 mmHg from baseline at hour 3, without an increase in the dose of background vasopressors. The trial was registered in the clinicaltrials.gov repository (no. NCT02338843) and conducted in accordance with current Good Clinical Practice guidelines and the ethical principles described in the Declaration of Helsinki.

### Patients

Patients enrolled in ATHOS-3 were ≥ 18 years of age with vasodilatory shock, MAP of 55–70 mmHg, despite adequate volume resuscitation and receipt of vasopressors at a dose > 0.2 µg/kg/min NED for 6–48 h prior to enrollment; background vasopressor use was not standardized and included catecholamines and vasopressin based on regional availability.

### Outcomes

In this post-hoc analysis, patients were grouped by the NED at study drug initiation into low (≤ 0.25 µg/kg/min) and high (> 0.25 µg/kg/min) subgroups. This cut-off was established on the basis of post-marketing observational studies demonstrating that 0.2–0.3 µg/kg/min NED is a common criterion in local hospital protocols for adding AT II [13], with one study suggesting better hemodynamic response to AT II below these thresholds [14]. Further, a study investigating the impact of NED on outcomes with vasopressin, reported that starting vasopressin at a NED dose of 10 µg/min was associated with a significantly lower likelihood of in-hospital mortality compared to waiting until a NED of 25 µg/min [17]. Presuming clinical use of AT II as part of an early multimodal approach along with NE (10 µg/min or 0.1 µg/kg/min) and vasopressin (0.04 units/min), would yield a NED of 0.2 µg/kg/min at initiation. We set our threshold slightly above this NED because of the ATHOS-3 eligibility criterion that patients be on a minimum NED of 0.2 µg/kg/min. Utilizing a NED cut-off criterion of 0.25 µg/kg/min resulted in an approximately 1:2 distribution of low: high-NED cases deemed suitable for this exploratory analysis. Finally, we performed sensitivity analyses using NED thresholds of 0.2 and 0.3 µg/kg/min.

Within each subgroup, we compared the differences in 28-day survival between patients receiving AT II or placebo. The primary outcome was the difference in 28-day survival between the AT II and placebo groups in those with a baseline NED ≤ 0.25 µg/kg/min at the time of study drug initiation. The secondary outcome was the difference in 28-day survival between the two groups in those with a baseline NED > 0.25 µg/kg/min. Other exploratory outcomes included the proportion of patients achieving a MAP response at hour 3, the degree of change in background vasopressors at hour 3, survival at 7 days, and cumulative incidence of discontinuation of renal replacement therapy (RRT) at 7 days. Safety was assessed and reported through 28 days.

### Statistical methods

Categorical data were reported as counts with percentages and analyzed with chi-square or Fisher exact test in univariate analysis, as appropriate. Continuous data were reported as medians with interquartile ranges and analyzed with Wilcoxon rank sum test in univariate analysis. Kaplan–Meier analyses were used to describe time-to-event variables including survival and cumulative incidence of RRT discontinuation. The log-rank test was used to compare the AT II and placebo arms with strata defined by the randomization strata [16]. Cox proportional hazards models were used to compare the AT II and placebo arms utilizing the same a priori defined methods as the ATHOS-3 clinical trial stratified by the randomization strata adjusted for MAP and APACHE II, with effects estimated by the hazard ratios (HR) [16]. A two sided α of 0.05 was used to test for differences in treatment outcomes without adjustment for multiplicity. Statistical analysis was performed with SAS version 9.4 (Cary, NC: SAS Institute Inc.).

## Results

### Baseline demographics

Of the 321 patients, 104 were initiated on study drug with a low-NED (≤ 0.25 µg/kg/min) and 217 with a high-NED (> 0.25 µg/kg/min) at baseline. The baseline demographics of AT II and placebo groups within the NED subgroups were well balanced, with the exception of significantly more females in the high-NED AT II subgroup compared to the high-NED placebo subgroup (Table 1). Severity of illness based on APACHE II and SOFA scores was similar across subgroups with the exception of statistically higher SOFA score in the high-NED placebo subgroup. Dysregulation of the RAAS was evident at baseline in both the low-NED and high-NED subgroups, however, there were no significant differences in the levels of any RAAS component between groups (Table 1). Further, significantly fewer patients were receiving RRT in the high-NED AT II subgroup.

**Table 1:** Baseline demographics

	Low-NED (≤ 0.25 µg/kg/min)		*p*-value	High-NED (> 0.25 µg/kg/min)		*p*-value
	Placebo (n = 48)	AT II (n = 56)		Placebo (n = 110)	AT II (n = 107)	
Age (yrs)	65.0 (50–75)	63.0 (53–73)	0.91	65.0 (53–75)	63.0 (51–75)	0.78
Female sex, n (%)	21 (43.8)	21 (37.5)	0.55	34 (30.9)	50 (46.7)	0.02
BMI (kg/m^2^)	31.0 (25.5–37.6)	28.8 (23.9–35.4)	0.18	28.4 (23.6–34.2)	28.1 (24.2–32.9)	0.62
Hypertension, n (%)	29 (60.4)	37 (66.1)	0.68	58 (52.3)	61 (56.5)	0.59
Exposure to ACE inhibitors, n (%)	6 (12.5)	6 (10.7)	1.00	9 (8.2)	9 (8.4)	1.00
Exposure to ARBs, n (%)	5 (10.4)	5 (8.9)	1.00	6 (5.5)	6 (5.6)	1.00
APACHE II score	27.0 (20–34)	27.0 (20–33)	0.81	29.5 (24–34)	27.0 (23–33)	0.14
SOFA score	12 (10–13)	12 (10–14)	0.55	13 (11–15)	12 (10–13)	< 0.001
Cardiac index (L/min/m^2^)*	3.2 (2.7–4.3)	3.0 (2.5–3.8)	0.25	3.2 (2.6–3.9)	3.0 (2.6–3.8)	0.68
Lactate (mmol/L)	2.2 (1.2–3.6)	2.4 (1.5–3.3)	0.67	4.1 (2.2–8.2)	3.1 (2.1–5.6)	0.07
Chest X-ray finding of ARDS, n (%)	13 (27.1)	11 (19.6)	0.48	38 (34.5)	29 (27.4)	0.30
MAP (mmHg)	67.3 (65.7–68.9)	67.5 (65.3–69.7)	0.94	65.7 (62.0–68.0)	65.7 (63.3–68.7)	0.28
NED (µg/kg/min)	0.21 (0.19–0.23)	0.21 (0.18–0.23)	0.45	0.45 (0.34–0.70)	0.47 (0.33–0.68)	0.75
Vasopressin use in the 6 h prior to randomization, n (%)^†^	30 (62.5)	34 (60.7)	1.00	81 (73.6)	79 (73.8)	1.00
Steroid use, n (%)	14 (29.2)	10 (17.9)	0.24	34 (30.9)	28 (26.2)	0.46
Receiving RRT, n (%)	9 (18.8)	17 (30.4)	0.26	54 (49.1)	31 (29.0)	0.003
Angiotensin I (pg/ml)	171.5 (64.0–446.0)	240.5 (55.8–558.0)	0.75	257.0 (77.9–828.0)	285.0 (87.4–869.0)	0.73
Angiotensin II (pg/ml)	80.5 (25.4–345.0)	72.1 (16.9–203.0)	0.41	64.9 (16.7–342.0)	116.0 (32.2–257.0)	0.33
Angiotensin I:II ratio	1.2 (0.7–2.9)	1.3 (0.8–7.4)	0.26	2.0 (1.1–5.8)	1.7 (1.0–4.8)	0.66
Renin (pg/ml)	65.6 (33.5–197.1)	138.8 (43.6–252.4)	0.23	285.8 (89.1–754.2)	153.0 (81.1–630.5)	0.20

Unless otherwise noted, all values are expressed as median (IQR)

*ARDS*, acute respiratory distress syndrome; *AT II*, angiotensin II; *BMI*, body mass index; *MAP*, mean arterial pressure; *NED*, norepinephrine-equivalent dose; *RRT*, renal replacement therapy

*n’s for cardiac index were: 15, 24, 58, and 45 for each subgroup, respectively. For the remaining subjects, the measures of central venous oxygen saturation of greater than 70% coupled with central venous pressure of greater than 8 mmHg were used to confirm vasodilatory shock. There were no significant differences between subgroups in either of these latter measures

^†^The use of vasopressin was not standardized and was based on the regional availability. The mean dose across subgroups was 0.04 U/min

### Hemodynamics and vasopressor administration

In both the low-NED and high-NED subgroups, patients randomized to receive AT II had a significantly higher MAP response compared to placebo (low-NED subgroup, 78.6% AT II vs. 25.0% placebo, *p* < 0.001; high-NED subgroup, 65.4% AT II vs. 22.7% placebo, *p* < 0.001). Also compared to placebo, patients receiving AT II experienced a greater magnitude MAP increase from baseline to hour 3 (low-NED subgroup, 11 mmHg AT II vs. 2 mmHg placebo, *p* < 0.001; high-NED subgroup, 11 mmHg AT II vs. 4 mmHg placebo, *p* < 0.001) (Table 2). The mean decrease in NED from baseline to hour 3 was significantly greater in patients receiving AT II in the low-NED subgroup compared to placebo (− 0.02 ± 0.06 µg/kg/min vs. − 0.01 ± 0.06 µg/kg/min; *p* = 0.01) and also in the high-NED subgroup compared to placebo (− 0.05 ± 0.12 µg/kg/min vs. 0.03 ± 0.31 µg/kg/min; *p* = 0.01) (Table 2).

**Table 2:** Hemodynamic, Vasopressor, and Exploratory Endpoint Data

	Low-NED (≤ 0.25 µg/kg/min)		HR (95% CI)	*p*-value	High-NED (> 0.25 µg/kg/min)		HR (95% CI)	*p*-value
	Placebo (n = 48)	AT II (n = 56)			Placebo (n = 110)	AT II (n = 107)		
MAP response at hour 3, n (%)	12 (25.0)	44 (78.6)		< 0.001	25 (22.7)	70 (65.4)		< 0.001
MAP change from baseline to hour 3, mmHg	2 (− 1–8)	11 (7–16)		< 0.001	4 (− 1–10)	11 (5–16)		< 0.001
NED at hour 3, µg/kg/min	0.21 (0.18–0.24)	0.19 (0.15–0.23)		0.01	0.46 (0.33–0.76)	0.42 (0.28–0.67)		0.20
NED change from baseline to 3 h, µg/kg/min (mean ± SD)	− 0.01 ± 0.06	− 0.02 ± 0.06		0.01	0.03 ± 0.31	− 0.05 ± 0.12		0.01
7-d survival, %	71 (56–82)	87 (76–94)	0.38 (0.15, 0.94)	0.03	63 (53–71)	63 (53–71)	0.99 (0.63,1.53)	0.94
7-d cumulative incidence of discontinuing RRT, %	33 (12–72)	59 (37–81)	1.99 (0.55–7.24)	0.29	11 (5–23)	29 (16–48)	2.97 (1.06–8.36)	0.03

Unless otherwise noted, all values are expressed as median (IQR)

*AT II*, angiotensin II; *MAP*, mean arterial pressure; *NED*, norepinephrine-equivalent dose; *RRT*, renal replacement therapy; *SD*, standard deviation

### Survival

In the placebo group, 25/48 (52%) patients in the low-NED subgroup and 60/110 (55%) patients in the high-NED subgroup had died at 28 days. In the AT II group, 20/56 (36%) patients in the low-NED subgroup and 55/107 (51%) in the high-NED subgroup had died by day 28 (Table 3). Among all patients receiving AT II, those randomized at low-NED were more likely to survive compared to high-NED at both 7 days (87% vs. 63%, *p* = 0.001) and 28 days (64% vs. 49%; *p* = 0.03). However, within the placebo group, no differences in survival were seen amongst those randomized at low-NED compared to high-NED at either day 7 (71% vs. 63%, *p* = 0.56) or at day 28 (48% vs. 45%; *p* = 0.98).

**Table 3:** Primary and secondary outcomes

*Primary outcome, 28-day survival in the Low-NED (*≤ *0.25 µg/kg/min) subgroup*			
Placebo (n = 48)	AT II (n = 56)	HR (95% CI)	*p*-value
48% (33%–61%)	64% (50%–75%)	0.51 (0.27, 0.95)	0.03

*Secondary outcome, 28-day survival in the High-NED (> 0.25 µg/kg/min) subgroup*			
Placebo (n = 110)	AT II (n = 107)	HR (95% CI)	*p*-value
45% (36–54%)	49% (39–58%)	0.93 (0.64, 1.35)	0.71

Values are expressed as median (IQR)

*AT II*, angiotensin II; *NED*, norepinephrine-equivalent dose

**Fig. 1:**
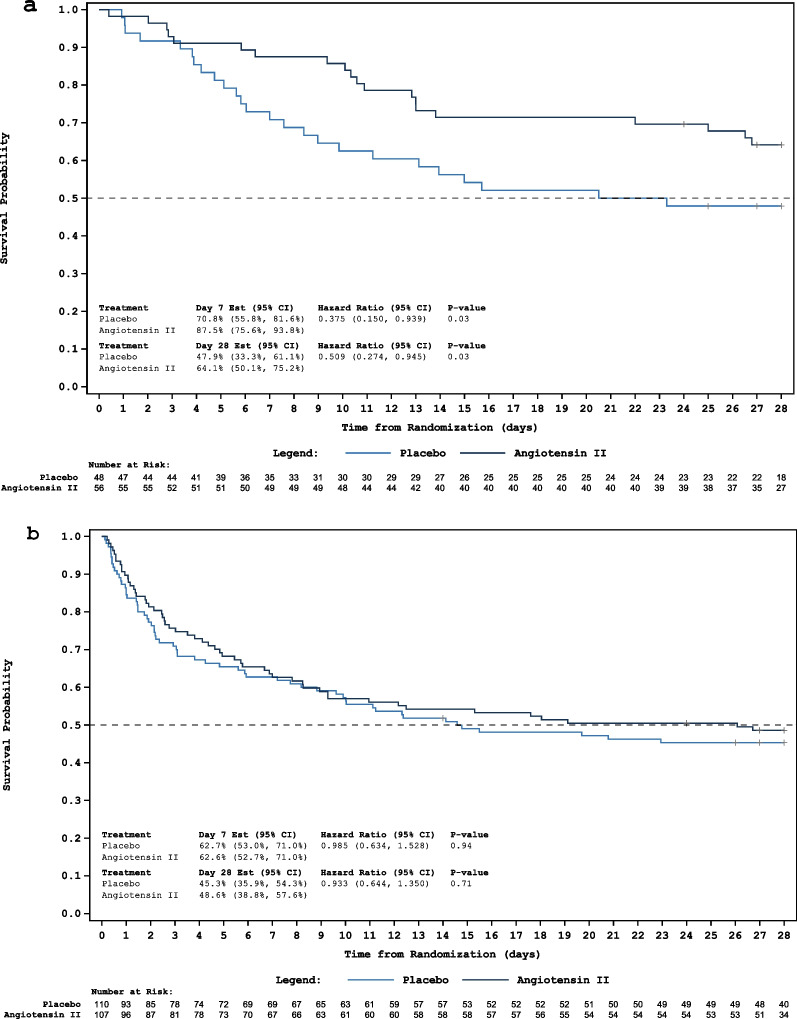
Kaplan–Meier plots of 28-day survival in patients randomized at low-NED (**a**) and in the high-NED arms (**b**)

In the low-NED subgroups, those randomized to AT II were significantly more likely to survive at 28 days compared to those randomized to placebo (HR 0.51; 95% CI 0.27–0.95, *p* = 0.03, Fig. 1a, Table 3, Additional file 1: Fig. S1). The absolute increase in survival of 16% with AT II resulted in a number needed to treat of 7. In the high-NED subgroups, no difference in 28-day survival between the AT II and placebo groups was found (HR 0.93; 95% CI 0.64–1.35, *p* = 0.71, Fig. 1b, Table 3, Additional file 1: Fig. S1). At 7 days, receipt of AT II in the low-NED subgroup was associated with significantly increased survival compared to placebo (HR 0.38; 95% CI 0.15–0.94, *p* = 0.03, Fig. 1a, Table 2). No difference in the rate of 7-day survival was again found in the high NED subgroup (Fig. 1b, Table 2). Additional file 1: Fig. S2 displays 28-day survival by baseline NED in all enrolled patients. Additional file 1: Fig. S3 displays 28-day survival by baseline NED according to treatment arm.

### Renal recovery of patients on RRT

Within the low-NED subgroup, renal recovery (defined as cumulative incidence of discontinuing RRT) at day 7 in patients receiving RRT at randomization in the AT II group was 59% compared to 33% in the placebo group (HR 1.99; 95% CI: 0.55–7.24; *p* = 0.29) (Table 2). In the high-NED subgroup, significantly more patients in the AT II group achieved renal recovery by day 7 (29%) as compared to patients in the placebo group (11%) (HR 2.97; 95% CI: 1.06–8.36; *p* = 0.03) (Table 2).

### Safety

In the low-NED subgroup, treatment-emergent adverse events were reported in 11.3% fewer patients randomized to AT II compared to placebo (*p* = 0.16), whereas in the high-NED cohort, these were only 1.1% lower in the AT II group (Table 4). Similarly, in the low-NED subgroup, numerically fewer patients receiving AT II compared to placebo experienced serious adverse events (48.2% vs. 66.7%, respectively; *p* = 0.07), but not in the high-NED cohort (67.3% AT II vs. 67.3% placebo).

**Table 4:** Summary of safety events

	Low-NED (≤ 0.25 µg/kg/min)		*p*-value	High-NED (> 0.25 µg/kg/min)		*p*-value
	Placebo (n = 48)	AT II (n = 56)		Placebo (n = 110)	AT II (n = 107)	
TEAE						
Events	194	212		368	457	
Patients, n (%)	44 (91.7)	45 (80.4)	0.16	101 (91.8)	97 (90.7)	0.81
SAE						
Events	51	53		116	118	
Patients, n (%)	32 (66.7)	27 (48.2)	0.07	74 (67.3)	72 (67.3)	1.0
SAE with frequency ≥ 4% in any study group, n (%)*						
Infections and infestations (any)	4 (8.3)	6 (10.7)		17 (15.5)	24 (22.4)	
Septic shock	1 (2.1)	1 (1.8)		9 (8.2)	17 (15.9)	
Nervous system disorders (any)	3 (6.3)	0 (0)		6 (5.5)	7 (6.5)	
Cardiac disorders (any)	10 (20.8)	10 (17.9)		22 (20.0)	17 (15.9)	
Atrial fibrillation	2 (4.2)	0 (0)		3 (2.7)	5 (4.7)	
Cardiac arrest	3 (6.3)	2 (3.6)		6 (5.5)	5 (4.7)	
Ventricular tachycardia	1 (2.1)	3 (5.4%)		2 (1.8)	2 (1.9)	
Vascular disorders (any)	3 (6.3)	4 (7.1)		12 (10.9)	13 (12.1)	
Respiratory, thoracic, and mediastinal disorders (any)	13 (27.1)	6 (10.7)		12 (10.9)	11 (10.3)	
Acute respiratory failure	5 (10.4)	0 (0)		0 (0)	3 (2.8)	
Respiratory failure	7 (14.6)	3 (5.4)		4 (3.6)	5 (4.7)	
Gastrointestinal disorders (any)	3 (6.3)	1 (1.8)		5 (4.5)	2 (1.9)	
Hepatobiliary disorders (any)	0 (0)	1 (1.8)		5 (4.5)	3 (2.8)	
Renal and urinary disorders (any)	3 (6.3)	0 (0)		0 (0)	2 (1.9)	
General disorders and administration site conditions (any)	7 (14.6)	11 (19.6)		18 (16.4)	16 (15.0)	
Multi-organ failure	6 (12.5)	10 (17.9)		17 (15.5)	15 (14.0)	

*AT II*, angiotensin II; *NED*, norepinephrine-equivalent dose; *SAE*, serious adverse event; *TEAE*, treatment-emergent adverse event

*For each event category, patients were counted once even if they had multiple events within that category. Adverse events were coded according to the Medical Dictionary for Regulatory Activities

### Sensitivity analysis

Sensitivity Analysis of the primary outcomes was performed at a NED threshold of 0.2 µg/kg/min and 0.3 µg/kg/min. At a threshold of 0.2 µg/kg/min, there were only 27 patients in the low-NED AT II group and 18 patients in the low-NED placebo group. Overall, with a sample size this low, there was not enough power to detect a difference in 28-day survival between these two groups (HR 0.55; 95% CI 0.19–1.55, *p* = 0.25). At a threshold of 0.3 µg/kg/min, there were a total of 139 patients in the low-NED group, and 182 patients in the high-NED group. Within the low-NED group, 74 patients received AT II and 65 patients received placebo. At this threshold, the difference in survival was not statistically significant (HR 0.62; 95% CI 0.36–1.07, *p* = 0.08).

## Discussion

This exploratory post-hoc analysis of the phase 3 ATHOS-3 clinical trial was undertaken to determine whether there is a signal for benefit of intervening with AT II at lower doses of other vasopressor agents. While this analysis is only hypothesis-generating in nature, we found a narrow window of low background vasopressor dose at which adding AT II may be associated with better hemodynamic response and increased survival in patients with vasodilatory shock. Specifically, more patients achieved the hemodynamic target with AT II when initiated at low-NED (≤ 0.25 µg/kg/min) compared to high-NED (> 0.25 µg/kg/min). Further, amongst patients with low-NED at the time of study drug initiation, those randomized to AT II were significantly more likely to survive at 7 and 28 days compared to those randomized to placebo, and within the AT II arm, those randomized at low-NED were significantly more likely to survive compared to those randomized at high-NED. Importantly however, this analysis cannot establish the true beneficial window for intervention with AT II, but suggests that it may occur at lower NED, and may also be narrow.

Vasodilatory shock is considered a medical emergency that requires hypotension-correcting interventions [4]. Catecholamines and vasopressin have traditionally been the initial vasopressors administered in patients with persistent hypotension in spite of fluid therapy [4]. However, these agents alone may be inadequate to achieve and maintain hemodynamic goals [5]; indeed, vasopressin achieves target hemodynamic goals in less than 50% of patients [18–20]. Escalation of vasopressor dosage in patients with persistent hypotension is thus common, and although dosing thresholds in outcomes research vary, exposure to high dosages of vasopressors has consistently portended grave prognosis in observational studies [21]. For example, shock unresponsive to norepinephrine or NED of ≥ 1 µg/kg/min is associated with a mortality rate > 80% [22, 23]. One retrospective study of 1610 patients with septic shock, reported that in-hospital mortality rose significantly for each 10 µg/min increase in NED at vasopressin addition (OR 1.21, 95% CI 1.09–1.34) [17]. In another observational study, 30-day mortality was greater amongst 270 patients when AT II was added at higher NED (HR 1.61, 95% CI 1.03–2.51, *p* = 0.037; per 1 µg/kg/min) [13]. In those who do survive exposure to high-dose vasopressors, such high doses provide diminishing hemodynamic benefits, and instead, increased harm manifests as arrhythmias and end-organ ischemia [21]. Our study corroborates previously reported data and supports the idea that addition of a non-catecholamine vasopressor at lower NED may be associated with improved survival. Therefore, the NED may identify patients with poor prognosis, and may be an important determinant of outcomes in shock and provide valuable insight into the optimal management strategies.

While unable to demonstrate any causal relationship, our post-hoc analysis suggests that if started at a lower NED, AT II may be associated with increased survival compared with placebo. Importantly, this analysis demonstrates that there may be a narrow range of NED at which intervention with AT II may be beneficial and above which may be futile. These results are similar to an observational study which found increased hemodynamic benefit with addition of AT II at NED < 0.3 µg/kg/min and even more pronounced benefit when the NED was lower (< 0.2 µg/kg/min) [14]. The present data, coupled with the well-established high mortality rates of high vasopressor dosages, suggest that in vasodilatory shock, addition of AT II may be more likely to achieve a better outcome if done so when the cumulative catecholamine vasopressor dosages are lower. Recently-published pilot data suggested that ATII may even have a role as a primary vasopressor in certain patients with distributive shock [24]. Similar findings have been demonstrated with vasopressin, wherein the addition of vasopressin at higher NED was associated with increased odds of in-hospital death (20.7% increase with every 10 µg/min increase in norepinephrine) [17]. Further, in a prospectively defined stratum of patients in the VASST trial, patients with septic shock randomized to vasopressin while receiving norepinephrine at a dose less than 15 µg/min were less likely to die at 90 days than patients receiving norepinephrine alone (35.8% vs. 46.1%; absolute risk reduction, − 10.4% [95% CI, − 20.3 to − 0.4%) [25].

Reasons for why the addition of AT II at lower NED may be associated with improved outcomes are likely multifactorial but may include NED sparing, RAAS-specific mechanisms, and potentially beneficial interaction between vasopressor agents with differing mechanisms of action. In the ATHOS-3 trial, the addition of AT II lowered the requirement for background vasopressors throughout the majority of the 48-h treatment period [7]. Further, a large observational study found that the catecholamine sparing effect of AT II was greatest when it was started at lower NED [14]. Because of the potential toxicity associated with high catecholamine load [22, 23, 26], these data suggest that NED sparing may contribute to the outcome differences observed in our study. This suggestion is further supported by the rates of serious adverse events in the current analysis, which, while not statistically significant, were nearly 20% lower in the low-NED AT II subgroup than in all other subgroups.

Additionally, RAAS disruption and AT II deficiency have been described to occur in septic shock and vasoplegic syndrome following cardiac surgery and to be significantly associated with adverse outcomes [10, 11, 27–32]. Hormonal repletion with AT II in patients with RAAS disruption may significantly improve survival [10]. While there was evidence of derangement in the concentrations of each component of the RAAS in this analysis (compared to normal reference ranges), there were no significant differences between subgroups and no clear trends in relationship to NED. Therefore, while AT I:II excess and hyper-reninemia may be an influencing factor on outcome in distributive shock [10, 11], determinants of outcomes in patients treated with AT II are likely multifactorial and may also include the overall severity and progression of the shock state, and thereby the vasopressor dose.

Finally, while we were unable in this post-hoc analysis to determine whether there is any interaction between vasopressor agents, there may be a potentiating effect of AT II and vasopressin. Such an interaction was suggested in an observational study of AT II, in which pre-existing use of vasopressin at the time of AT II initiation was associated with a significantly higher rate of hemodynamic responsiveness to AT II (OR 6.05, 95% CI 1.98–18.6; *p* = 0.002) [13]. However, 30-day mortality was not impacted by the use of vasopressin in that study (OR 1.32, 95% CI 0.70–2.48; *p* = 0.39), though few patients (22/270, 8.1%) were not receiving vasopressin at the time of intervention with AT II. Future trials may investigate whether there is any potentiating effect of vasopressin and AT II, and whether intervening with both agents at low norepinephrine doses may be more beneficial than intervening with either alone.

The present analysis, although hypothesis-generating in scope, has potentially important clinical implications, as it adds to mounting evidence suggesting that the NED may be an important determinant of outcomes in shock and could be used to guide management strategies. The current Surviving Sepsis Campaign guidelines do not provide specific recommendations regarding timing for initiation of non-catecholamine vasopressors in patients with persistent hypotension, but the guideline authors state that their typical approach is to start vasopressin at a norepinephrine dose of 0.25–0.5 µg/kg/min [4]. Our analysis, together with a similar recent analysis with vasopressin [17], suggests instead that a multimodal strategy initiated at a lower norepinephrine dose may be warranted. The current analysis further suggests that an earlier multimodal therapeutic approach utilizing AT II may confer benefit [33], though future prospective trials are necessary to confirm this.

The current analysis has several strengths, including the use of data from a prospective randomized trial of patients with vasodilatory shock. Further, the analysis addresses an important clinical question, the most appropriate scenario for intervention with AT II, which is not currently defined. Finally, the present findings are consistent with those from other analyses suggesting that interventions with non-catecholamine agents at lower norepinephrine doses are associated with improved outcomes [14, 17].

We acknowledge several limitations. First, this is a post-hoc observational study of a phase 3 trial with a non-patient-centered primary outcome. Thus, no inferences of causality of AT II on the patient-centered outcomes reported herein can be drawn; the results should be considered exploratory, hypothesis-generating, and the interpretation of the results requires caution. Second, the study design of ATHOS-3 resulted in only one third of patients being enrolled at NED ≤ 0.25 µg/kg/min, limiting the generalizability of these data. Had the ATHOS-3 protocol allowed for earlier enrollment, additional analyses could have been conducted on this population of patients. However, the limited number of patients enrolled in ATHOS-3 below this threshold preclude further analysis of thresholds below 0.25 µg/kg/min. Further, the total number of patients in each subgroup was small and so the findings are subject to type I error. Third, it is possible that low-NED and high-NED do not correlate with early or late stage of disease. Disease trajectory may be such that low-NED may not necessarily be early in the shock state but could potentially be a sign of patient improvement. Therefore, no conclusions regarding timing can be drawn from this analysis. Finally, even within the placebo group, practice may have been variable with regard to vasopressors [34], which may have influenced the results.

## Conclusions

This exploratory post-hoc analysis of a phase 3 clinical trial suggests that the introduction of AT II therapy at lower doses of vasopressor therapy may be associated with benefit. Prospective randomized trials of AT II infusion in lower NED patients may be both desirable and justified. While the data presented herein should be considered exploratory, hypothesis-generating, and should be interpreted with caution, they may inform selection of an appropriate threshold vasopressor dose below which introduction of AT II could be investigated.

## Supplementary Information

**Additional file 1: Fig. S1.** Kaplan-Meier plots of 28-day survival in patients randomized at low-norepinephrine equivalent dose and high-norepinephrine equivalent dose separated by treatment arms. **Fig. S2.** Day 28 survival by baseline norepinephrine equivalent dose in all enrolled patients. **Fig. S3.** Day 28 survival by baseline norepinephrine equivalent dose separated by treatment group. Blue: angiotensin II, red: placebo.

## Data Availability

The data supporting the findings of this study are available from La Jolla Pharmaceutical Company. However, restrictions apply to the availability of these data, which were used under license for the current study and so are not publicly available. However, data are available from the authors upon reasonable request and with the permission of La Jolla Pharmaceutical Company.

## Abbreviations

AT II: Angiotensin II
ATHOS-3: Angiotensin II for the treatment of high-output shock
HR: Hazard ratio
MAP: Mean arterial pressure
NED: Norepinephrine-equivalent dose
RAAS: Renin–angiotensin–aldosterone system
