# Supplementary material for: Initiating angiotensin II at lower vasopressor doses in vasodilatory shock: an exploratory post-hoc analysis of the ATHOS-3 clinical trial
Source: Crit Care. 2023 May 5;27:175. doi: 10.1186/s13054-023-04446-1 (PMC10163684; doi:10.1186/s13054-023-04446-1)

SUPPLEMENTAL MATERIAL

**Figure S1. Kaplan-Meier plots of 28-day survival in patients randomized at low-norepinephrine equivalent dose and high-norepinephrine equivalent dose separated by treatment arms.**

**
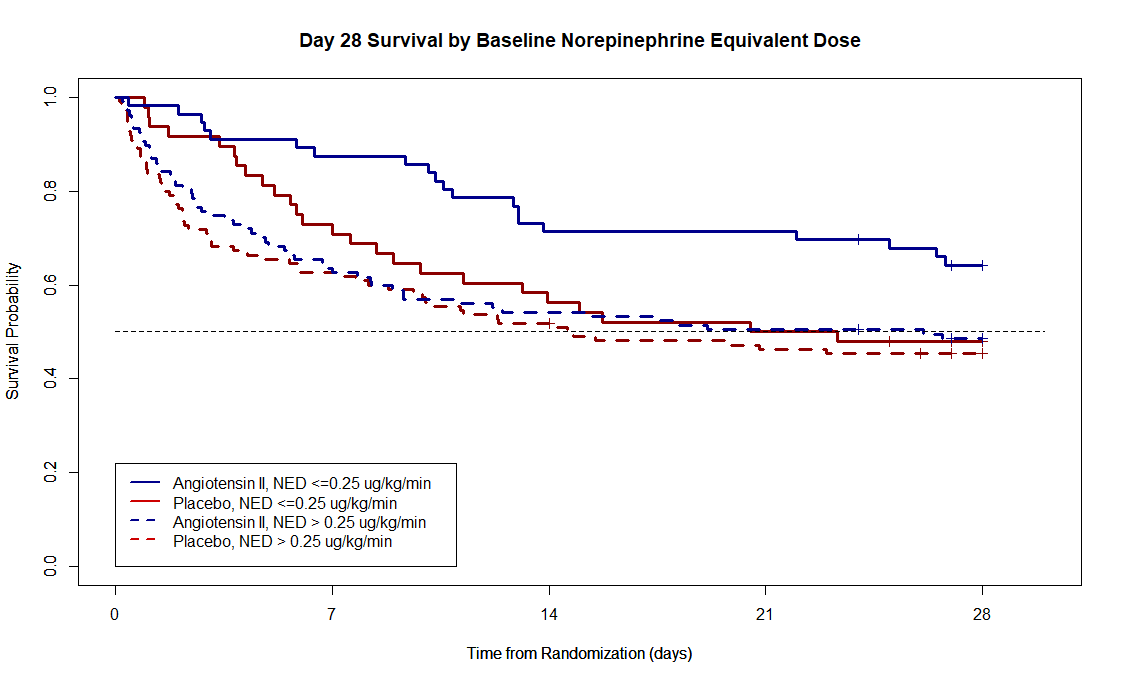
**

**Figure S2. Day 28 survival by baseline norepinephrine equivalent dose in all enrolled patients.**


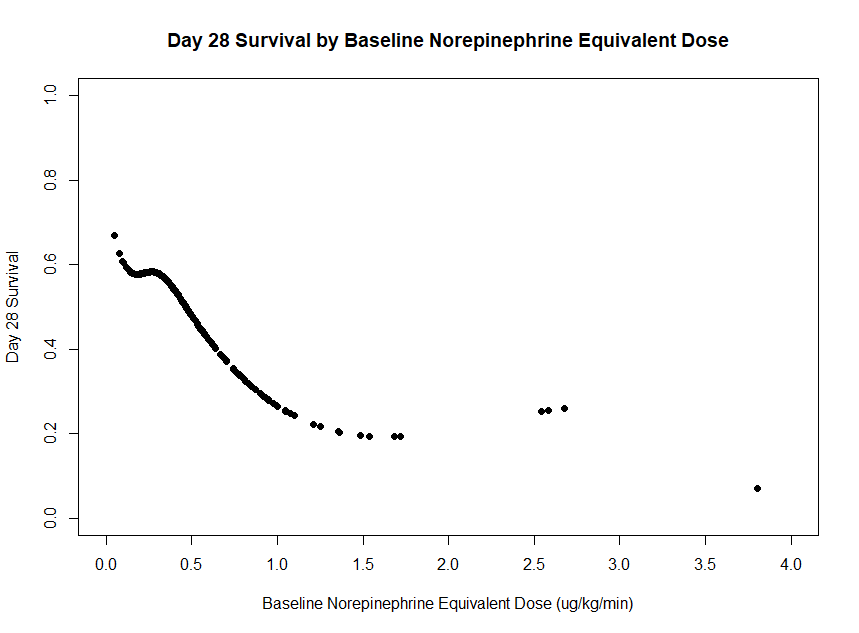


**Figure S3. Day 28 survival by baseline norepinephrine equivalent dose separated by treatment group. Blue: angiotensin II, red: placebo.**


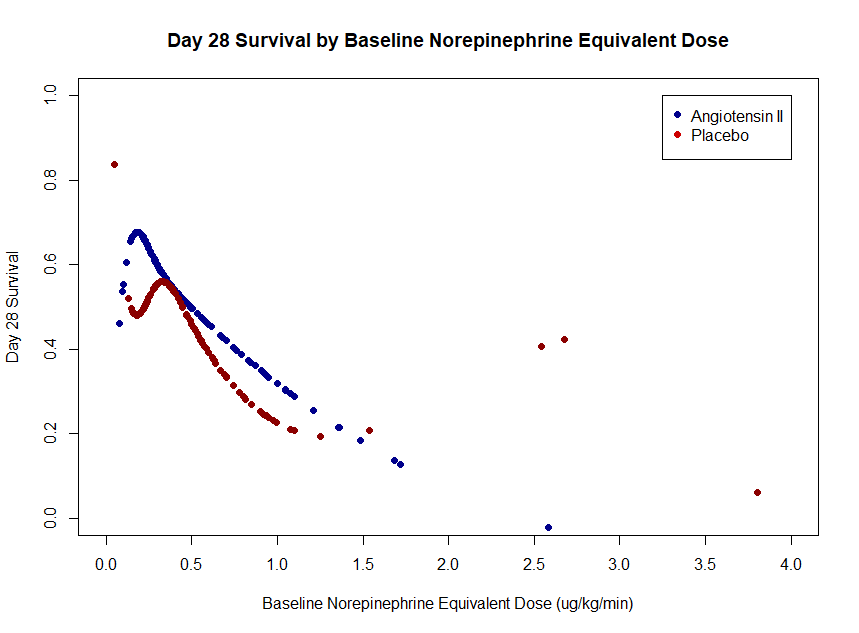

Supplement: Supplementary file 1 — Additional file 1: Fig. S1. Kaplan-Meier plots of 28-day survival in patients randomized at low-norepinephrine equivalent dose and high-norepinephrine equivalent dose separated by treatment arms. Fig. S2. Day 28 survival by baseline norepinephrine equivalent dose in all enrolled patients. Fig. S3. Day 28 survival by baseline norepinephrine equivalent dose separated by treatment group. Blue: angiotensin II, red: placebo. [file 13054_2023_4446_MOESM1_ESM.docx]
